# Conjunctival lymphoma arising from reactive lymphoid hyperplasia

**DOI:** 10.1186/1477-7819-10-194

**Published:** 2012-09-18

**Authors:** Junichi Fukuhara, Satoru Kase, Mika Noda, Kan Ishijima, Teppei Yamamoto, Susumu Ishida

**Affiliations:** 1 Department of Ophthalmology, Hokkaido University Graduate School of Medicine, Nishi 7, Kita 15, Kita-ku, Sapporo, 060-8638, Japan

**Keywords:** Conjunctiva, Extra nodal marginal zone B-cell lymphoma, Reactive lymphoid hyperplasia

## Abstract

Extra nodal marginal zone B-cell lymphoma (EMZL) of the conjunctiva typically arises in the marginal zone of mucosa-associated lymphoid tissue. The pathogenesis of conjunctival EMZL remains unknown. We describe an unusual case of EMZL arising from reactive lymphoid hyperplasia (RLH) of the conjunctiva. A 35-year-old woman had fleshy salmon-pink conjunctival tumors in both eyes, oculus uterque (OU). Specimens from conjunctival tumors in the right eye, oculus dexter (OD), revealed a collection of small lymphoid cells in the stroma. Immunohistochemically, immunoglobulin (Ig) light chain restriction was not detected. In contrast, diffuse atypical lymphoid cell infiltration was noted in the left eye, oculus sinister (OS), and positive for CD20, a marker for B cells OS. The tumors were histologically diagnosed as RLH OD, and EMZL OS. PCR analysis detected IgH gene rearrangement in the joining region (JH) region OU. After 11 months, a re-biopsy specimen demonstrated EMZL based on compatible pathological and genetic findings OD, arising from RLH. This case suggests that even if the diagnosis of the conjunctival lymphoproliferative lesions is histologically benign, confirmation of the B-cell clonality by checking IgH gene rearrangement should be useful to predict the incidence of malignancy.

## Background

Extra nodal marginal zone B-cell lymphoma (EMZL) of the conjunctiva typically arises in the marginal zone of mucosa-associated lymphoid tissue. EMZL is common among malignant conjunctival tumors and has a quite indolent course, but relapses can be seen [1]. The pathogenesis of conjunctival EMZL, however, remains unknown. Herein, we report an unusual case of EMZL arising from reactive lymphoid hyperplasia (RLH) of the conjunctiva. 

**Figure 1:**
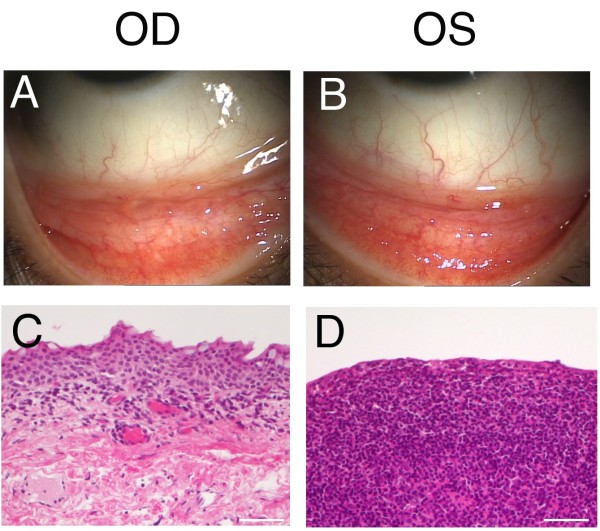
**Slit-lamp (A, B) and histological (C, D) examinations at the initial presentation.** Slit-lamp examination reveals a conjunctival tumor with a salmon-pink appearance (A, B). Histological examination demonstrates a collection of small lymphoid cells in the stroma OD (C, H & E staining). In contrast, diffuse atypical lymphoid cell infiltration is noted, involving the conjunctival epithelium and stroma OS (D, H & E staining). Bar indicates 100 μm. OD, oculus dexter; OS, oculus sinister.

**Figure 2:**
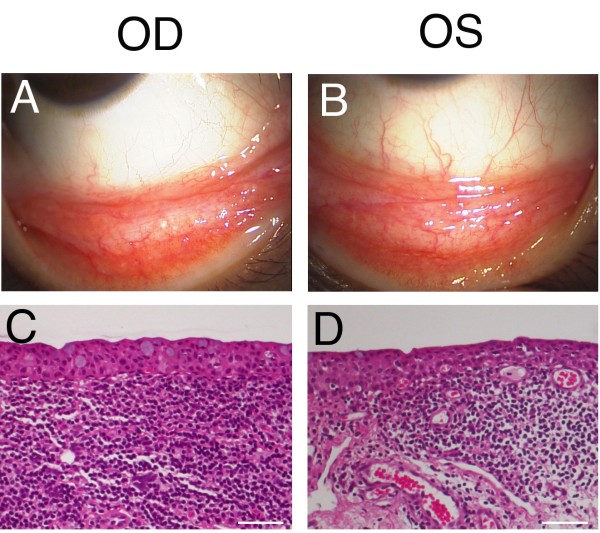
**Slit-lamp (A, B) and histological (C, D) examinations 11 months after the initial presentation.** The salmon-pink conjunctival tumor remains unchanged OD (A). The conjunctival tumor shows marginal regression after radiotherapy OS (B). Histologically, diffuse atypical lymphoid cell infiltration with plasma cells is observed OD (C, H & E staining), while small lymphoid cells infiltrate the stroma OS (D, H & E staining). Bar indicates 100 μm. OD, oculus dexter; OS, oculus sinister.

## Case presentation

A 35-year-old woman had suffered from minor irritation in both eyes, oculus uterque (OU), and was treated with topical eye drops based on a diagnosis of allergic conjunctivitis at an initial clinic. Since her conjunctival lesions did not improve, she was referred to our hospital on 17 December 2010. Her visual acuity was 20/20 OU with a normal intraocular pressure. Slit-lamp examination demonstrated fleshy salmon-pink tumors in the upper and lower conjunctival fornix that extended into the inferior palpebral conjunctiva (Figure 1A,B ). The fundus was normal. Laboratory data and complete blood counts were within normal limits. No systemic abnormality was detected except for the conjunctivas. Biopsy of the inferior conjunctival tumors was performed OU.

Formalin-fixed, paraffin-embedded serial tissue sections were cut at a 4 μm thickness, and dewaxed paraffin sections were stained with H & E staining. The sections were also submitted for immunohistochemistry. After preparation of dewaxed paraffin sections, endogenous peroxidase activity was inhibited by immersing the slides in 3% hydrogen peroxide in methanol for 10 minutes. Then, non-specific binding of the primary antibody was blocked by incubating the slides in blocking bovine serum for 30 minutes. The slides were incubated with primary antibodies, consisting of anti-CD20, CD3, immunoglobulin G (IgG) kappa and IgG lambda antibodies (Dako, Carpinteria, CA, USA), at room temperature for two hours. Positive signals were visualized using diaminobendizine as a substrate.

Histological examination demonstrated a collection of small lymphoid cells in the stroma (Figure 1C) OD. Immunohistochemically, Ig light chain restriction was not detected. In contrast, diffuse atypical lymphoid cell infiltration was noted together with lymphoepithelial lesions OS (Figure 1D). Immunohistochemically, atypical lymphoid cells were positive for CD20, a marker for B cells. The tumors were histologically diagnosed as RLH OD, and EMZL OS. After the extraction of DNA from the fleshy unfixed conjunctival tumor tissues, PCR analysis detected Ig heavy chain (IgH) gene rearrangement in VH(FR1)/JH region OD and VH(FR2)/JH region OS. Radiotherapy with a total dosage of 30 Gy was administered OS. Slit-lamp examination demonstrated a less marked change of the conjunctival tumor OD (Figure 2A), whereas the tumor volume marginally regressed OS after radiation (Figure 2B). Biopsy of the inferior conjunctival tumors was performed again on 27 October 2011. A histological examination showed diffuse atypical lymphoid cell infiltration, admixed with plasma cells OD (Figure 2C). Immunohistochemically, the atypical lymphoid cells were positive for CD20 with deviation to kappa chains of Ig in the infiltrating lymphoid cells. Isolated DNA from the second biopsy tissue revealed IgH gene rearrangement in VH(FR1)/JH and VH(FR2)/JH regions OD. Therefore, a diagnosis of EMZL was made, arising from RLH OD. In contrast, small lymphoid cells infiltrated the stroma OS (Figure 2D), where IgH gene rearrangement was not detected.

## Discussion

In this case, the diagnosis of EMZL was made OS, based on typical histological findings and B-cell monoclonality with IgH gene rearrangement. In contrast, histological findings revealed RLH OD, although IgH gene rearrangement was detected using the PCR method. After 11 months, a re-biopsy specimen demonstrated EMZL based on compatible pathological and genetic findings OD. Previous studies reported that RLH of the orbit and eyelid developed systemic lymphomas in the selected cases [2,3]; however, there was no report showing EMZL of the conjunctiva which arose from RLH *in situ*.

While EMZL had been thought to be induced by infection and/or chronic inflammation, the pathogens have yet to be determined. EMZL may arise at sites of chronic antigenic stimulation due to autoimmunity (for example, Sjogren sialadenitis [4]) but the etiology remains unknown. We recently reported a bilateral conjunctival lymphoproliferative disorder presenting with diffuse lymphoid cell infiltration with IgH gene rearrangement in one eye and without it in the contralateral eye [5]. These results suggested that conjunctival lymphoma might arise from RLH.

Conjunctival lymphatic tissue develops after antigen stimulation [6]. Chronic antigen stimulation basically initiates a reactive lymphoid infiltrate with polyclonality in the normally sterile conjunctival tissues. In contrast, in some inflammatory situations, reactive clonal proliferations of B cells can occur in response to antigenic stimulation [7], which may be observed during the development of conjunctival lymphatic tissue. In this case, the histopathology initially demonstrated mild small lymphoid cell infiltration in the stroma OD, where IgH gene rearrangement was detected, indicating reactive clonal proliferation of B cells in the conjunctival tissue. After 11 months, EMZL was eventually detected in the same eye. These processes suggest that the B-cell clonal expansion plays a potential role in the pathogenesis of malignancy from the benign conjunctival RLH, which contributes to the increased incidence of lymphoma in association with this disorder, as seen in sialadenitis [8]. This case suggests that even if the diagnosis of the conjunctival lymphoproliferative lesions is histologically benign, confirmation of the B-cell clonality by checking IgH gene rearrangement should be useful to predict the incidence of malignancy.

## Conclusion

The results in this case suggest that conjunctival lymphoma may arise from reactive lymphoid hyperplasia. This case suggests that even if the diagnosis of the conjunctival lymphoproliferative lesions is histologically benign, confirmation of the B-cell clonality by checking IgH gene rearrangement should be useful to predict the incidence of malignancy.

## Abbreviations

EMZL: extra nodal marginal zone B-cell lymphoma; H & E: haematoxylin and eosin; Ig: immunoglobulin; OD: oculus dexter (right eye); OS: oculus sinister (left eye); OU: oculus uterque (both eyes); PCR: polymerase chain reaction; RLH: reactive lymphoid hyperplasia.

## Competing interests

The authors have no proprietary interests in any aspect of this study.

## Authors’ contributions

JF, SK and MN proposed the study. JF and SK obtained images, wrote the manuscript provided and reviewed the pathological images. SI, KI and TY conducted the literature search. All authors read and approved the final manuscript.
